# In Vitro Fertilization in Africa: Mild Versus Conventional Antagonist Ovarian Stimulation Protocols in Expected Normal Responders Undergoing IVF/ICSI: A Cohort Study From Ethiopia

**DOI:** 10.1002/puh2.70089

**Published:** 2025-07-28

**Authors:** Mekia Kedir, Mustafa Negash, Abel Teshome, Abraham Fessehaye Sium

**Affiliations:** ^1^ Department of Obstetrics and Gynecology St. Paul's Hospital Millennium Medical College (SPHMMC) Addis Ababa Ethiopia

**Keywords:** ART, in vitro fertilization (IVF), low‐middle income, ovarian stimulation, Sub‐Saharan

## Abstract

**Background:**

Though infertility remains a global public health problem, it stands as a unique challenge in Sub‐Saharan Africa, with either no or limited in vitro fertilization (IVF) service in most countries located in it. This study aimed to compare the efficacy of mild stimulation with conventional GnRH antagonist ovarian stimulation in patients with expected normal responders at a public IVF center in Ethiopia.

**Methods:**

A retrospective cohort study was conducted at Center for Fertility and Reproductive Medicine in Ethiopia. The primary outcomes were biochemical pregnancy rate, clinical pregnancy rate, and mature (M2) oocyte retrieval rate. Data were collected retrospectively by reviewing medical records of patients. SPSS version 26 was used to analyze the data. Mann–Whitney *U* test and chi‐squared/exact were performed as appropriate. *p* value less than 0.05 was used to present the results significance.

**Results:**

Five hundred infertile women (age ≤39) undergoing their first IVF cycle. A total of 150 women exposed to mild stimulation group and 350 women exposed to conventional or antagonist IVF‐group). The biochemical pregnancy was higher in MS‐IVF, which is 35.3% and 28.6% in conventional (antagonist) IVF group, *p* value = 0.04. Clinical pregnancy rate (35.4% in the MS‐IVF vs. 25.8% in the antagonist arm, *p* value = 0.2) and early abortion (8.8% in the MS‐IVF group vs. 3.7% in the antagonist group, *p* value = 0.3) were not statistically different between the groups. There was no difference in the rate of ovarian hyperstimulation syndrome (OHSS) between the groups. The total dose of gonadotrophin used for stimulation was lower in MS group (1274 ± 546 in MS group vs. 2776 ± 719 in the antagonist group, *p* < 0.001).

**Conclusions:**

Compared to conventional antagonist IVF, MS‐IVF has a higher biochemical pregnancy and lower risk of multiple pregnancy with comparative rates of clinical pregnancy, OHSS, and mature oocyte (M2).

## Graphical abstract

A cohort study was conducted on IVF outcomes using different IVF protocols: 150 women exposed to mild stimulation (MS) IVF protocol were compared to matching 350 women exposed to conventional or antagonist IVF protocol. We found MS‐IVF has higher biochemical pregnancy and lower risk of complications compared to conventional IVF.

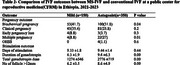

## 1 Introduction

In recent years, there has been an increasing interest in developing new, milder approaches in in vitro fertilization (IVF) treatment that might decrease the physical burden and psychological distress for patients, increase patient convenience, and contribute to reducing treatment costs [1]. Mild stimulation IVF is defined as a protocol in which the ovaries are stimulated with lower dose gonadotrophins, usually <150 IU, and/or other pharmacological compounds to limit the number of oocytes following stimulation for IVF [2]. The International Society for Mild Approaches in Assisted Reproduction (ISMAAR) committee recommends adopting a mild approach to ovarian stimulation in all clinical settings as an increasing body of evidence suggests that MS is as effective as conventional stimulation [3, 4, 5]. Multiple studies show that mini‐IVF results in high pregnancy rates, low multiple pregnancy rates, low ovarian hyperstimulation syndrome (OHSS) rates, and low cost [6, 7]. However, the majority of the literature on this topic stems from high‐income countries, with no prior studies that documented the effectiveness of this protocol in the Sub‐Saharan Africa, which has one of the highest burdens of infertility but with virtually absent IVF services in most countries in this region of Africa.

In Ethiopia, public IVF center was introduced in 2019, and this public facility has been vital in addressing the advanced treatment needs of infertile couples from Ethiopia and beyond from the continent of Africa. Ever since, thousands of IVF procedures have been performed, and majority of them are performed by senior reproductive endocrinology and infertility (REI) specialists. At the center, IVF is provided using different IVF protocols: conventional long‐agonist, antagonist, or minimal stimulation protocols with pure recombinant follicle‐stimulating hormone (FSH). Our study sought to compare the efficacy of mild stimulation (minimal) with conventional GnRH antagonist ovarian stimulation in patients with expected normal responders at this public IVF center in Ethiopia.

## 2 Methods

### 2.1 Study Design, Setting, and Population

This study was a retrospective cohort study on IVF outcomes when minimal stimulation (MS‐IVF) protocol was used compared to when conventional IVF protocol was used over 2 years period (from Aug 2021 to July 2023) at St. Paul's Hospital Millennium Medical College (Ethiopia). St. Paul's Hospital is a leading tertiary hospital in Ethiopia with several sub‐specialty care and trainings available within the college, including REI. The hospital has the only public center for reproductive medicine (CFRM) in the Horn of Africa with several hundreds of IVF performed at this center since its introduction in 2017. We conducted the study by assigning the study participants into cohorts: minimal stimulation IVF group and conventional IVF group. The primary outcomes of this study were to compare the rates of biochemical pregnancy, clinical pregnancy, and mature oocyte (M2) retrieval between minimal stimulation (MS‐IVF) and conventional (antagonist) IVF. We considered this outcome instead of live birth rate of a singleton pregnancy, which is the recommended one, mainly due to the reason that most of the patients who receive IVF care continue their prenatal care and delivery services in different centers—including private hospitals (as the only public IVF center in East Africa, our center hosts many referral‐in patients from around Ethiopia and East African countries). Biochemical pregnancy was assessed on the basis of a positive pregnancy test (blood or urine), but the pregnancy is not viable enough to be seen on ultrasound. We assessed clinical pregnancy based on ultrasound findings in addition to blood tests and gestational sacs seen at 4–6 weeks. The secondary outcomes were ovarian hyperstimulation rate, multiple pregnancy risk, dose of gonadotropin used, and total oocyte count. We assessed hyperstimulation based on the presence of symptoms like abdominal pain, bloating, and nausea, along with ultrasound evidence of enlarged ovaries and fluid accumulation in the peritoneum. Multiple pregnancies were assessed on the basis of early obstetric ultrasound findings.

Inclusion criteria were patients undergoing COH with expected to have normal response with AFC >5. The exclusion criteria were patients with repeated IVF trial and high responders (AFC >18) and those with incomplete data.

### 2.2 IVF Protocols

In the long protocol, GnRH agonists were given during the luteal phase of the menstrual cycle, and ovarian stimulation using gonadotropins was started on Days 2 or 3 of the menses. In the conventional (antagonist protocol), ovarian stimulation was started using gonadotropins on Days 2 or 3 of menses, and the GnRH antagonist would be started once the dominant ovarian follicle reached a size of 14 mm. For the mild stimulation, we used a modification of the antagonist protocol where the ovarian stimulation was started on the second day of menses using an oral aromatase inhibitor (letrozole) or clomiphene citrate and stimulation with gonadotropins was initiated on the fourth day. The woman would then be started on antagonist medication when the dominant follicle reaches a size of 14 mm.

### 2.3 Data Collection Procedure

Data were collected on Open Data Kit (ODK) by reviewing medical records of patients. A structured data collection questionnaire prepared in English was used to collect the data, and data were collected by midwives trained on the data collection. We collected socio‐demographic data, infertility clinical characteristics, IVF protocols along with their outcomes.

### 2.4 Sample Size Calculation and Sampling Procedure

The sample size was determined using sample size calculation for cohort study by taking p1 = 14.6% and p2 = 30.9% for positive clinical pregnancies in a similar study done previously [8]. The ratio of matching cohorts to cases (mild stimulation—MS‐IVIF) was 5/2. The final sample size for MS‐IVF was n1 = 150, and for the matching cohorts (conventional IVF‐antagonist group), n2 = 350. A total of 600 study subjects were included in the study. Non‐random sampling technique was used to recruit study participants.

### 2.5 Data Processing and Analysis

Data were entered and analyzed using IBM SPSS version 26 statistical software. Simple descriptive statistics were used to analyze the baseline characteristics. Chi‐squared test/Fisher exact and Mann–Whitney test were employed to compare the primary and secondary outcomes of the study between the study groups. *p* value less than 0.05 was used to present the findings significance.

## 3 Results

A total of 500 women were included in the final analysis. Among them, 350 women were treated with the GnRH antagonist protocol (antagonist group), whereas 150 women received mild protocol (MS‐IVS group). The distribution of baseline characteristics between both groups (MS‐IVS and antagonist groups) (Table 1) was similar, including clinical variables that can influence IVF outcome, such as age, AFC, cause of infertility, and type of infertility—primary versus secondary.

**TABLE 1:** Baseline characteristics of patients who had in vitro fertilization (IVF) with MS IVF and conventional (antagonist) IVF protocols at a public center for reproductive medicine in Ethiopia, 2021–2023 (*n* = 500).

	Mild stimulation (MS) (*n* = 150)	Antagonist (*n* = 350)	
Variable	*N* (%)	*N* (%)	*p* value
Age (mean in years ± SEM)	31 ± 5.3	32 ± 4.9	0.07
Type of infertility			
Primary infertility	116 (77)	270 (77.1)	0.9
Secondary infertility	34 (22.7)	80 (22.9)	
Cause of infertility			
Tubal factor	76 (50.7)	168 (48)	0.6
Male factor	36 (24)	76 (21.7)	
Ovulatory	9 (6)	32 (9.1)	
Unexplained	25 (16.7)	58 (16.6)	
Combined	4 (2.7)	16 (4.6)	
Address			
Addis Ababa	84 (56)	235 (67.1)	0.1
Amhara region	16 (10.7)	27 (7.7)	
Oromia region	38 (25.3)	52 (14.9)	
SNNPR region	6 (4)	18 (5.1)	
Other	6 (4)	18 (5.1)	
AFC (mean in IU/L ± SEM)	7.8 ± 3.0	8.5 ± 3.4	0.06

Abbreviations: AFC, antral follicular count; SEM, standard error mean.

When it comes to the IVF outcomes (Table 2), biochemical pregnancy (positive serum b‐HCG) was higher in MS‐IVF, which is 35.3% and 28.6% in antagonist group, with *p* value of 0.04. Clinical pregnancy rate (35.4% in the MS‐IVF vs. 25.8% in the antagonist arm, *p* value = 0.2) and early abortion (8.8% in the MS‐IVF group vs. 3.7% in the antagonist group, *p* value = 0.3) were not statistically different between the groups. There was no difference in the rate of OHSS between the groups. However, multiple pregnancy rates were higher in conventional IVF group (antagonist) than in the MS‐IVF group (27% vs. 8.8%, *p* value = 0.01). The total dose of gonadotrophin used for stimulation was lower in MS group (1274 ± 546 vs. 2776 ± 719 in the antagonist group, *p* < 0.001), though total number of stimulation days was similar between the groups (9.33 ± 1.8 in the MS‐IVF group vs. 9.44 ± 1.4 in the antagonist group, *p* value = 0.44). Type of IVF, embryo type, good embryo quality, and number of fertilizations were similar between the groups.

**TABLE 2:** Comparison of in vitro fertilization (IVF) outcomes between MS‐IVF and conventional IVF at a public center for reproductive medicine (CFRM) in Ethiopia, 2021–2023.

	Mild (*n* = 150)	Antagonist (*n* = 350)	
Outcome	*N* (%)	*N* (%)	*p* value
**Pregnancy outcome**			
Biochemical pregnancy	53 (35.3)	100 (28.6)	0.04
Clinical pregnancy	45 (35.4)	81 (25.8)	0.2
Early pregnancy loss	4 (8.8)	3 (3.7)	0.3
Multiple pregnancy	4 (8.8)	22 (27)	0.01
OHSS	0	4 (1.1)	0.6
**Stimulation outcome**			
Stimulation duration (mean in ± SEM)	9.33 ± 1.8	9.44 ± 1.4	0.44
Duration of gonadotropin (mean ± SEM	6.3 ± 1.9	9.4 ± 6.3	0.00
Total gonadotropin dose (mean ± SEM)	1274 ± 546	2776 ± 719	0.00
No. of follicle >12 mm (mean ± SEM)	6.2 ± 3.5	8.6 ± 4.9	0.00
Cancelled cycle	10 (6.7)	4 (1.1)	0.001
Endometrial thickness (mean ± SEM)	9.49 ± 2.2	8.8 ± 2.2	0.002
No. of retrieved oocyte (mean ± SEM)	6.9 ± 4.7	9.1 ± 6.0	<0.001
No. of M2 oocytes (mean ± SEM)	4.1 ± 2.4	6.2 ± 5	0.3
No egg	4 (2.9)	12 (3.5)	0.7
No fertilization	13 (9.3)	32 (9.2)	0.5
Good quality embryo	101 (67.3)	227 (64.9)	0.3
**Type of fertilization**			0.2
IVF	48 (34.3)	140 (40.5)	
ICSI	92 (65.7)	206 (59.5)	
Embryo type			0.5
Fresh	113 (80.7)	267 (77.2)	
Frozen	14 (10)	47 (13.6)	

Abbreviation: SEM, standard error mean.

## 4 Discussion

In this study, compared to conventional antagonist IVF, MS‐IVF has a higher biochemical pregnancy and lower risk of multiple pregnancy with comparative rates of clinical pregnancy, OHSS, and mature oocyte (M2) but with lesser risk of multiple pregnancy. Furthermore, the dose of gonadotropin used for stimulation in the conventional group was more than two times that utilized in the minimal stimulation (MS‐IVF protocol).

Currently, mild ovarian stimulation (minimal stimulation) stands as a cheaper and patient‐friendly alternative to the conventional IVF (antagonist) protocol. It has also been mentioned as a treatment option for poor responders in IVF treatment [8, 9, 10, 11]. Despite similar rates of clinical pregnancy and M2 retrieval between the conventional (antagonist) protocol and MS‐IVF protocol in our study, the finding of higher biochemical pregnancy rate in the MS‐IVF protocol in our study supports this growing evidence regarding MS‐IVF protocol. In a large systematic review and meta‐analysis, it was found no difference in live birth rate (*n* = 1057, RR 0.91, CI 0.66–1.25) and ongoing pregnancy (*n* = 1782, RR 1.01, CI 0.86–1.20) between mild and conventional IVF protocols. Furthermore, the number and proportion of high‐grade embryos were similar. Mild IVF also resulted in reduced use and cost [9].

Among the important findings of our study, MS‐IVF protocol had a reduced multiple pregnancy rate with no encounter of OHSS and a much lower dose of gonadotropin was used compared to conventional IVF protocol. This implies increased safety of IVF for patients with the MS‐IVF protocol, a favorable outcome of utmost importance during IVF, and a cheaper cost of IVF, as the cost of drugs (gonadotropins) constitute a bigger percentage of expenses for the IVF care, especially in low‐income settings. However, whether this lower cost of MS‐IVF would motivate infertile women to undergo more than one MS‐IVF cycle for nearly similar price as a single conventional IVF cycle should be further explored in future studies that should include risk‐benefit analysis, including patient acceptability of the protocols.

Being among the first to present information on the benefits of minimal stimulation (MS‐IVF) protocol from a low‐income setting, our study adds a very useful information to current literature on this topic. Lack of randomization, retrospective nature of the data, and not being a multi‐center study are the main limitations of our study. Not including analysis of BMI, duration of sub‐fertility, and ovarian reserve is the other limitation of our study, as these could potentially bias the outcomes.

## 5 Conclusion

Compared to conventional IVF, MS‐IVF has higher biochemical pregnancy and lower risk of multiple pregnancy with comparative rates of clinical pregnancy and OHSS. Importantly, MS‐IVF protocol reduces gonadotropin consumption (cost) by more than a half, compared to the conventional protocol. Overall, our findings are in favor of the use of MS‐IVF protocol over conventional protocol, especially in low‐income settings, though patient acceptability of the protocols along with cost–benefit analysis should be further explored with well‐designed multi‐center studies.

## Author Contributions

Mekia Kedir and Mustafa Negash contributed to conception and development of the study protocol. Mekia Kedir, Mustafa Negash, and Abel Teshome contributed to data collection. Mekia Kedir performed the data analysis. Abraham Fessehaye Sium and Mekia Kedir contributed data interpretation and manuscript write‐up. Abraham Fessehaye Sium edited the final manuscript. All authors critically revised the article for intellectual content. All authors reviewed the final manuscript and approved its submission for publication.

## Ethics Statement

Formal ethical clearance letter was obtained from institutional review board of St. Paul Hospital Millennium Medical College with a reference PM23/304. The ethical clearance did not require obtaining informed consent from study subjects; hence, it was not obtained from the participants of this study.

## Conflicts of Interest

The authors declare no conflicts of interest.

## Data Availability

All data generated or analyzed during this study is included in this published article.

## References

[1] K. Kato , Y. Takehara , T. Segawa , et al., “Minimal Ovarian Stimulation Combined With Elective Single Embryo Transfer Policy: Age‐Specific Results of a Large, Single‐Centre, Japanese Cohort,” Reproductive Biology and Endocrinology: RB&E 10 (2012): 35. 22541043 10.1186/1477-7827-10-35 PMC3407520

[2] F. Zegers‐Hochschild , G. D. Adamson , J. De Mouzon , et al., “The International Committee for Monitoring Assisted Reproductive Technology (ICMART) and the World Health Organization (WHO) Revised Glossary on ART Terminology, 2009,” Human Reproduction 24, no. 11 (2009): 2683–2687. 19801627 10.1093/humrep/dep343

[3] ESHRE Guideline Group on Ovarian Stimulation , E. Bosch , S. Broer , et al., “ESHRE Guideline: Ovarian Stimulation for IVF/ICSI,” Human Reproduction Open 2020, no. 2 (2020): hoaa009. 32395637 10.1093/hropen/hoaa009 PMC7203749

[4] G. Nargund , “ISMAAR: The International Society for Mild Approaches in Assisted Reproduction,” Facts, Views & Vision in ObGyn 3, no. 1 (2011): 5–7.

[5] G. Nargund , A. K. Datta , S. Campbell , et al., “The Case for Mild Stimulation for IVF: Recommendations From The International Society for Mild Approaches in Assisted Reproduction,” Reproductive Biomedicine Online 45, no. 6 (2022): 1133–1144. 36220713 10.1016/j.rbmo.2022.07.019

[6] J. Lefebvre , R. Antaki , I. J. Kadoch , et al., “450 IU Versus 600 IU Gonadotropin for Controlled Ovarian Stimulation in Poor Responders: A Randomized Controlled Trial,” Fertility and Sterility 104 (2015): 1419–1425. 26361207 10.1016/j.fertnstert.2015.08.014

[7] J. J. Zhang , M. Feret , L. Chang , M. Yang , A. C. Badiola , and M. Van Wely , “A Randomized Clinical Trial Comparing Clomid Minimal Stimulation IVF to Conventional IVF,” Fertility and Sterility 102, no. 3 (2014): e23.

[8] C. Siristatidis , K. Dafopoulos , T. Vrantza , et al., “Mild versus Conventional Antagonist Ovarian Stimulation Protocols in Expected Normal Responders Undergoing IVF/ICSI: A Case‐Control Study,” Gynecological Endocrinology 33, no. 7 (2017): 553–556. 28277113 10.1080/09513590.2017.1296128

[9] A. K. Datta , A. Maheshwari , N. Felix , S. Campbell , and G. Nargund , “Mild versus Conventional Ovarian Stimulation for IVF in Poor Responders: A Systematic Review and Meta‐Analysis,” Reproductive Biomedicine Online 41, no. 2 (2020): 225–238. 32546333 10.1016/j.rbmo.2020.03.005

[10] A. K. Datta , A. Maheshwari , N. Felix , S. Campbell , and G. Nargund , “Mild Versus Conventional Ovarian Stimulation for IVF in Poor, Normal and Hyper‐Responders: A Systematic Review and Meta‐Analysis,” Human Reproduction Update 27, no. 2 (2021): 229–253. 33146690 10.1093/humupd/dmaa035 PMC7902993

[11] A. Revelli , S. Casano , F. Salvagno , and L. Delle Piane , “Milder is Better? Advantages and Disadvantages of,” Reproductive Biology and Endocrinology 9, no. 1 (2011): 1–9. 10.1186/1477-7827-9-25 PMC3048523 21324155

